# Assessment of chitosan-based edible coatings containing bioactive compounds derived from agricultural residue for improving postharvest quality characteristics of tomato (*Solanum lycopersicum* L.)

**DOI:** 10.3389/fnut.2025.1673029

**Published:** 2025-11-03

**Authors:** Sujeeta Yadav, Kamla Malik, Kashish Sharma, Pushpa Dhillon, Dandu Harikarthik

**Affiliations:** ^1^Department of Microbiology, College of Basic Sciences and Humanities, CCS Haryana Agricultural University, Hisar, India; ^2^Department of Botany and Plant Physiology, College of Basic Sciences and Humanities, CCS Haryana Agricultural University, Hisar, India

**Keywords:** rice straw, wheat straw, bioactive compounds, tomato, edible coating, shelf life

## Abstract

This study aimed to evaluate the impact of bioactive-based edible coatings on the shelf life of tomatoes. Bioactive compounds were extracted from rice and wheat straw. Different concentrations of phenolic extracts (0.2–1.0 g/mL) were blended with 1% chitosan and applied to fresh tomatoes stored at 28 °C and 74%−84% relative humidity (RH) for 30 days. Periodic evaluations revealed that tomatoes coated with 1.0 g/mL extract of rice and wheat straw coatings were highly effective in maintaining tomato quality as compared to controls. Tomatoes coated with 1.0 g/mL extract of wheat straw exhibited the most favorable results, including delayed weight loss (1.29%), slowed ripening, reduced pH levels, and lower lycopene (2.79 mg/100 g) and beta-carotene (0.62 mg/100 g) contents as compared to those coated with coatings containing rice straw extracts. Additionally, wheat straw extract-coated tomatoes had the lowest disease incidence (2%) after 30 days, as compared to 100% incidence in control samples. Overall, using edible coatings enriched with rice and wheat straw extracts presents a promising approach to extending the shelf life of tomatoes while preserving their nutritional value, inhibiting microbial growth, and offering a more sustainable and eco-friendlier alternative to conventional packaging methods.

## Graphical Abstract

**Figure:**
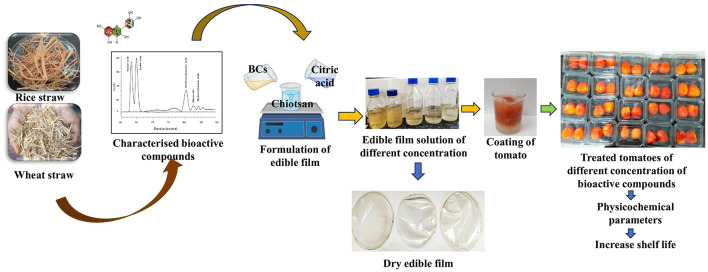

## Highlights

- Wheat straw extract (1.0 g/mL) coating reduced weight loss to 1.29 % and minimized ripening in tomatoes during 30 days of storage.
- Coated tomatoes retained lower lycopene (2.79 mg/100 g) and beta-carotene (0.62 mg/100 g), indicating better nutritional preservation.
- Edible coatings enriched with bioactive compounds from rice and wheat straw extracts were successfully developed, offering an eco-friendly solution for extending the shelf life of tomatoes.
- Tomatoes coated with a higher concentration of bioactive extracts showed significantly lower disease occurrence and severity compared to control samples.

## 1 Introduction

Currently, agriculture faces the dual challenge of meeting the increasing global demand for food production while adopting sustainable practices to minimize waste generated from agricultural activities. Over the past 50 years, the global population has increased from 3.7 billion to 7.9 billion and is projected to reach 8.6 billion by 2030, 9.8 billion by 2050, and 11.2 billion by 2100, according to a United Nations report (1). This rapid population growth has driven the need for increased agricultural output, which, in turn, has resulted in the generation of sustainable amounts of agricultural waste (2). Such waste contains valuable polyphenolic compounds, including flavonoids, tannins, phenolic acids, and anthocyanins, which offer potential for reuse in sustainable technologies such as edible coatings and films. Utilizing these compounds presents a promising solution for extending the shelf life and maintaining the quality of fruits and vegetables during postharvest storage. In recent times, consumers have become more worried about their food habits, rejecting products with additives and giving preference to fresh ones. Therefore, there is a dire need to develop a biodegradable edible coating for prolonging the shelf life of fruits and vegetables and preserving the nutritional quality of the fruit by minimizing the degradation of vitamins and antioxidants, potentially making them a sustainable alternative to traditional packaging methods.

Tomato (*Solanum lycopersicum* L.) is one of the most widely cultivated and significant crops, ranking second only to potatoes in annual global production (3). Despite being a rich source of vitamins and antioxidants, tomatoes are highly perishable, with a typical shelf life of 4 to 8 days at room temperature. Their rapid spoilage is primarily due to pathogenic infections, leading to significant postharvest losses (4, 5). Traditional packaging materials, such as polyethylene and polyvinyl chloride, have been widely used to mitigate these losses (6). However, these non-biodegradable materials pose significant environmental challenges, prompting increased interest in sustainable alternatives (7). Recently, biodegradable packaging materials have gained attention for their eco-friendly characteristics (8, 9). Edible coatings, composed of materials classified as Generally Recognized as Safe (GRAS), offer a promising solution for extending the shelf life of perishable produce (10). Among these, edible films, primarily composed of proteins or polysaccharides, have emerged as a promising solution, with polysaccharide-based packaging materials receiving particular focus (11–13). Among polysaccharide-based coatings, chitosan stands out for its favorable properties, including film-forming ability, biodegradability, antimicrobial activity, and antioxidant capacity (14, 15). However, its limitations, such as water sensitivity and poor mechanical strength, necessitate the incorporation of bioactive compounds such as polyphenols and compatible plasticizers to overcome this limitation by increasing the free volume and molecular mobility within the amorphous polymer matrix, thereby reducing intermolecular hydrogen bonding interactions and improving film flexibility (16, 17).

Agricultural wastes such as rice straw and wheat straw are rich in phenolic compounds with antioxidant and antimicrobial properties. Utilizing these extracts in chitosan-based films can reduce respiration rates, limit oxidative damage, and inhibit microbial growth, making them highly effective for extending the shelf life of tomatoes (18). Considering the present issue of the short shelf life of tomato fruits, this study aimed to develop and evaluate chitosan-based edible coatings enriched with phenolic extracts from agricultural waste to improve the postharvest quality and shelf life of *Hisar Arun* tomatoes, providing a sustainable alternative to conventional preservation methods. The objective of this study was to analyze the physiochemical (physiological loss, color, firmness, pH, and titrable acidity) and nutritional (ascorbic acid, lycopene, and β-carotene content) parameters of tomato during different intervals of storage.

## 2 Material and methods

### 2.1 Materials

Agricultural waste, such as rice straw and wheat straw, was collected from fields of CCS Haryana Agricultural University, Hisar, Haryana, India. All chemicals used in the study were of analytical grade and purchased from Thermo Scientific (Rockford, IL, USA). Fresh fruits of tomato (Hisar Arun) at the light red stage of ripening were procured from the farm of the Department of Vegetable Sciences, CCS Haryana Agricultural University, Hisar (Haryana) at 11:00 a.m. and transported in perforated plastic crates under ambient field temperature conditions (~30 °C).

### 2.2 Methods

Dried powder of rice and wheat straw was pretreated with NaOH (1.5%) and 5% H_2_SO_4_ at 90 °C with continuous stirring for 2 h as described by Kim and Han (78). Ultrasound-assisted extraction (UAE) (Vibra Cell^TM^ VCX750, Sonics & Materials, Inc., Newtown, CT, USA) was used for extraction of bioactive compounds from pretreated rice and wheat straw using 80% methanol as a solvent under ultrasonic field conditions at 40 °C for 55 min (maintaining the sample in an ice bath to prevent heating), using 750 W power, 20 kHz frequency, and 50% sonication amplitude. The solvent was further removed using a rotary evaporator (86, 89). The extracted bioactive compounds were dehydrated using anhydrous sodium sulfate and stored at 4 °C until further use.

#### 2.2.1 Preparation bioactive compound-based coating solution

To prepare the chitosan solution, 1% chitosan was dissolved in a 0.5% aqueous citric acid solution to enhance solubility. The solution was homogenized at 10,000 rpm for 10 min and stirred continuously for 60 min at room temperature (25 ± 2 °C) using a magnetic stirrer (Accumax, Neuation, iStir HP 10M). Next, 5 mL of rice straw (RS) and wheat straw (WS) extracts, with concentrations of 0.2 g/mL, 0.4 g/mL, 0.6 g/mL, 0.8 g/mL, and 1.0 g/mL (Table 1), along with 1 % glycerol as a plasticizer, were added to the prepared chitosan solution. This mixture was stirred for another 60 min at room temperature (25 ± 2 °C). The resulting solution was then used to prepare an edible coating.

**Table 1:** Formulation of chitosan-based edible coating enriched with phenolic extract.

**Coating formulations**	**Chitosan mL (v/v)**	**Phenolic extract (5%) (v/v)**
Control	0	0
RS1	95	0.2 g/mL
RS2	95	0.4 g/mL
RS3	95	0.6 g/mL
RS4	95	0.8 g/mL
RS5	95	1.0 g/mL
WS1	95	0.2 g/mL
WS2	95	0.4 g/mL
WS3	95	0.6 g/mL
WS4	95	0.8 g/mL
WS5	95	1.0 g/mL

RS, rice straw; WS, wheat straw.

#### 2.2.2 Coating of bioactive-based film on Hisar Arun tomatoes

**Figure 1:**
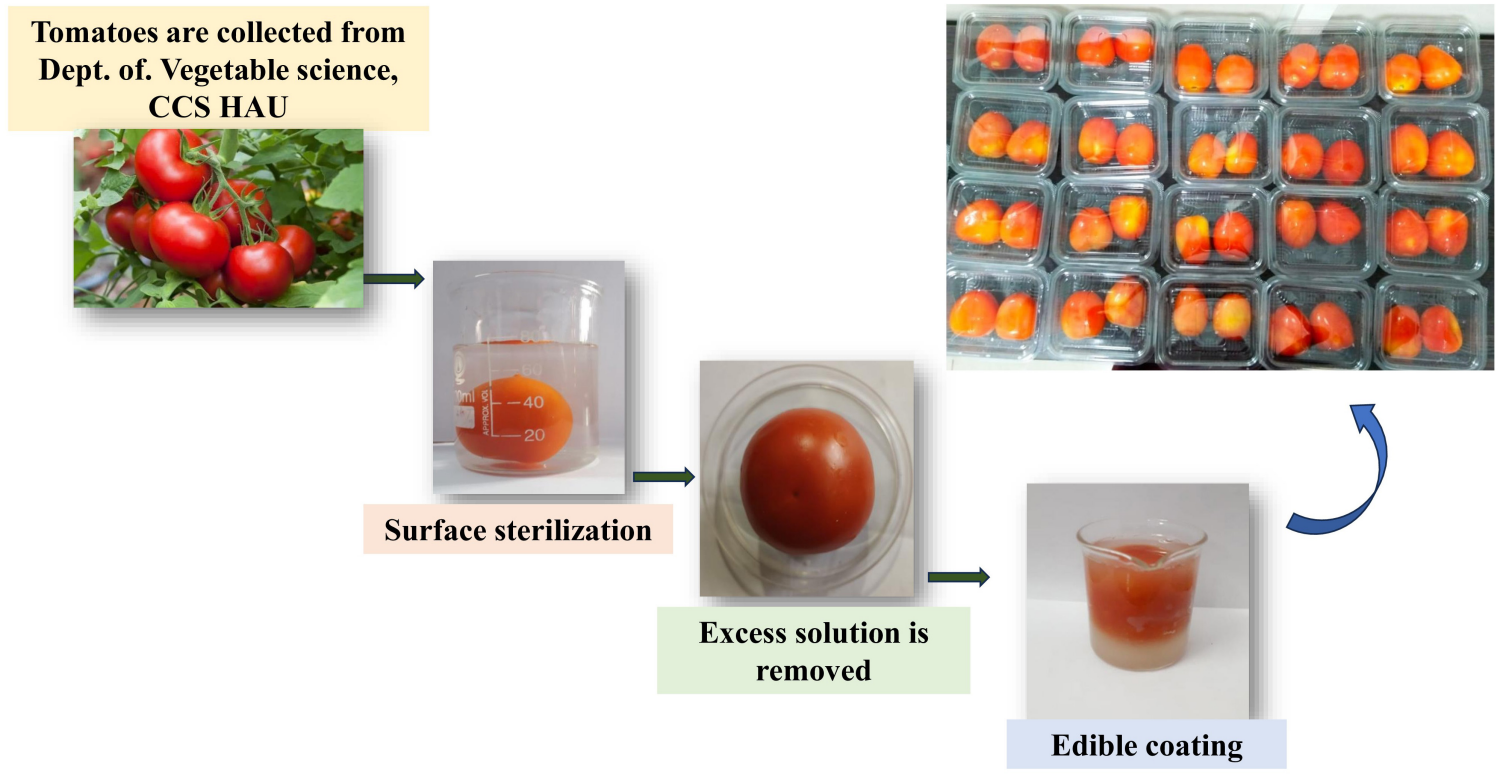
Schematic representation of bioactive compound-based coating on tomato.

Uniform-sized tomatoes were selected for coating. Initially, tomatoes were rinsed with sodium hypochlorite solution (50 ppm) for 1 min; excess sodium hypochlorite was removed by rinsing with distilled water. The fruits were coated using the immersion method as described by Mhd Haniffa et al. (19). A total of 10 formulations were prepared using rice and wheat straw extracts at concentrations of 0.2, 0.4, 0.6, 0.8, and 1.0 g/mL, along with an uncoated control (C), all maintained at room temperature (25 ± 2 °C). Three tomatoes per sample were used, and each treatment was conducted in three replications during the experiment. Fresh tomatoes were dipped into the coating solution for 1 min, allowed to drain, and air-dried at room temperature to remove excess coating. This process was repeated three times. The tomatoes were then allowed to dry at 25 °C, forming a thin-film coating (Figure 1). After drying, they were placed in polypropylene plastic covers and stored at room temperature. The coated tomatoes were weighed and stored at 25 ± 2 °C and 75 ± 5% relative humidity (RH) for 30 days.

### 2.3 Effect of coating on physicochemical parameters of tomatoes during the storage period

Various physicochemical parameters were analyzed during storage at 25 ± 2 °C and 75 ± 5% RH. Fruit weight loss, color, firmness, pH, and titrable acidity were analyzed at intervals, specifically on days 0, 7, 15, and 30. In addition, the incidence and severity of diseases were assessed on days 15 and 30 of storage to monitor the development of postharvest diseases.

#### 2.3.1 Weight loss

Tomato samples (three fruit per replication) were weighed at day 0 and the end of days 7, 15, and 30 of storage. The difference between the initial and final weight of the fruit was considered as total weight loss during the storage interval and calculated as percentages on a fresh weight basis by the standard (74) method. The results were reported as a weight loss percentage using the following formula:


$$\%\text{ Weight loss}=\;\frac{\text{Initial weight }-\text{ Final weight}}{\text{Initial weight}}\times 100$$

#### 2.3.2 Change in fruit firmness

The firmness of Hisar Arun tomatoes was evaluated using a handheld fruit pressure tester penetrometer (model BGS - 25 Make Biogen Scientific), equipped with a cylindrical plunger of 8 mm diameter and a firmness scale of 13 kg cm^−2^. To ensure accurate measurements, firmness was assessed on both sides of the equatorial region of each fruit. A total of three tomatoes were tested, with four random measurements taken on each tomato. The firmness of two tomatoes per treatment was measured, and it was expressed in kg cm^−2^. The average of these measurements was recorded and expressed in units of kg cm?^2^.

#### 2.3.3 Change in color and hue angle

The change in color of treated and untreated tomato samples was evaluated using a high-quality colorimeter (BCM-200). The L^*^, a^*^, b^*^ color spaces were employed to evaluate the impact of coatings on color change. The following parameters were recorded: L^*^ value (lightness), ranging from 0 (black) to 100 (white); an a^*^ value ranging from negative (green) to positive (red); and b^*^ value ranging from negative (blue) to positive (yellow). The color of tomatoes on day 0 was used as the reference point, and subsequent color changes were compared to this baseline. To ensure accurate and representative color measurements, multiple surface readings were taken from each sample by randomly repositioning the tomato fruits (20). To obtain the real color change during storage, a^*^ and b^*^ values were evaluated to calculate the hue angle value using the following equation (21):


$${\text{Hue}}^{\text{0}}=\text{arctg}\frac{{\text{b}}^{*}}{{\text{a}}^{*}}$$

where Hue^0^ = 0 represents purple red, 90^0^ represents yellow, 180^0^ represents green–blue, 270^0^ represents blue.

#### 2.3.4 Change in pH

Initially, the fresh tomatoes were juiced using an electric juicer, and then the resulting liquid was filtered through filter paper to remove any solids. The pH of the filtered tomato juice was then measured using a digital pH meter (Eutech, pH 700, Thermo Fisher Scientific) at storage intervals of 0, 7, 15, and 30 days to monitor changes over time. Each sample was analyzed in triplicate.

#### 2.3.5 Change in titrable acidity

The change in total acidity of tomato pulp during storage was determined using titration with 0.1 N NaOH, according to the method described by Ranganna (22). A total of 5 g of fruit pulp was macerated in 5 mL of distilled water, and the mixture was diluted to 100 mL with distilled water. The solution was thoroughly shaken and filtered through Whatman No. 1 filter paper to remove any solids. An aliquot of 20 mL filtrate was titrated with 0.1 N NaOH using 1% phenolphthalein as the indicator, and the endpoint was noted by the appearance of a stable pink color. The acidity of the sample was calculated based on the volume of NaOH used and expressed as a percentage (%).


$$\text{Acidity }(\%)\text{=}\frac{\text{Titre vol}.\;\left(\text{mL}\right)\times \text{Normality of alkali }\times \text{eq}.\text{ wt}.\text{ of acid }\times \text{vol}.\text{ made }(\text{mL})\times \text{100}}{\text{Vol}.\text{ of aliquot }\left(\text{mL}\right)\times \text{Wt}.\text{ of volume of sample }(\text{g})\times \text{1000}}$$

### 2.4 Effect of coating on the composition of bioactive compounds of tomatoes during storage

#### 2.4.1 Change in lycopene content

One gram of fresh tomato fruit was extracted twice with 10 mL of an acetone:n-hexane mixture (4:6 ratio). To facilitate extraction, the mixture was allowed to stand in an ice bath for 10 min, followed by centrifugation at 1,370 × *g* for 10 min. The supernatant was then separated using a separating funnel. The absorbance of the hydrophobic fraction was measured spectrophotometrically at wavelengths of 663, 645, 505, and 453 nm using a UV Vis spectrophotometer (Thermo Scientific™ GENESYS™ series, 180), with acetone as a blank (72). Three replicates were performed for each fruit sample. The lycopene concentration was quantified using the equation proposed by Nagata and Yamashita (23):


$$\begin{array}{r} {}[\text{Lycopene }(\text{mg}/100\text{mL})=-0.0458\;{\text{A}}_{663}+0.204\;{\text{A}}_{645}\\ +0.372\;{\text{A}}_{505}-0.0806\;{\text{A}}_{453}] \end{array}$$

#### 2.4.2 Change in β-carotene contents

The concentration of beta-carotene was determined using a colorimetric assay developed by Biswas et al. (24). A total of 500 mg of dried fresh tomato fruits was extracted twice with 5 mL of chilled acetone. The mixture was allowed to stand in an ice bath for 15 min with shaking and then mixed vigorously for 10 min. Subsequently, the mixture was centrifuged at 1,370 × g for 10 min. The resulting supernatants were collected and filtered using Whatman No. 1 filter paper, and their absorbance was measured at wavelengths 663, 645, 505, and 453 nm using a UV-Vis spectrophotometer (Thermo Scientific™ GENESYS™ series, 180). Each sample was analyzed in triplicate. The beta-carotene content was calculated using the following equation:


$$\begin{array}{r} {}[\beta -\text{carotene }(\text{mg}/100\text{mL})=-0.216\;{\text{A}}_{663}+1.22\;{\text{A}}_{645}\\ -0.304\;{\text{A}}_{505}+0.452\;{\text{A}}_{453}] \end{array}$$

#### 2.4.3 Change in ascorbic acid

The ascorbic acid content was determined using the method of Mukherjee and Choudhuri (25), which was based on the reduction of 2,4-dinitrophenyl hydrazine. A 0.1 mL aliquot of the sample was properly diluted and then mixed with 1.9 mL distilled water, 1 mL 2,4-dinitrophenyl hydrazine (2%), and a drop of 10% thiourea. The mixture was thoroughly mixed and kept in a boiling water bath for 15 min. After boiling, the mixture was cooled at room temperature, and the absorbance was measured at 530 nm using a UV-Vis spectrophotometer (Thermo Scientific™ GENESYS™ series, 180). The quantity of ascorbic acid was determined by comparing the absorbance reading to a standard curve of ascorbic acid, using concentrations ranging from 10 to 100 μg.

### 2.5 Disease incidence (DI)

The proportion of tomatoes affected by diseases is referred to as disease incidence. DI was determined as a percentage of fruit having symptoms of diseases such as dots and rots in each batch of storage (5). The percentage of disease incidence of tomatoes was calculated by using the following formula


$$\text{Disease incidence }\left(\text{DI}\right)\text{=}\frac{\text{number of tomatoes infected}}{\text{total number of tomatoes}}\times 100$$

### 2.6 Disease severity

Tomato disease severity (DS) was assessed following the method described by Mohamed et al. (26). The severity scale is used as follows: 0 = 0% (no visible symptoms); 1 = 1%−25% (fruit surface slightly necrotic spots and fungal mycelia); 2 = 26%−50% (fruit covered by necrotic spots and fungal mycelia); 3 = 51%−75% (presence of spore mass); and 4 = >75% (fruit appears soft and decayed).

### 2.7 Statistical analysis

The experimental data were statistically analyzed using analysis of variance techniques (ANOVA), using Duncan's multiple range test following the completely randomized design (CRD) method with the OPSTAT software available on the CCS HAU homepage (http://www.opstat.somee.com/). To visualize the results, heatmap and contour maps of representative data were constructed using R 2019 [3.3.2 (64-bit)] and Origin 2018 (64-bit) software. These analytical tools facilitated a more precise presentation and interpretation of the data by elucidating underlying patterns and variations within the results (82).

## 3 Results and discussion

### 3.1 Effect of coating on physiological weight loss of tomatoes

The percentage of weight loss during storage was affected by the maturity stage and biological variances of fruits (5). The different concentrations of extract used in coating treatments significantly affect the loss of weight of tomatoes over the 30 days of storage. Tables 2, 3 show the average weight loss of control and treated tomatoes with different concentrations of rice straw extract and wheat straw extract. After 30 days of storage, the percentage of weight loss was maximum in the control as compared to the treated samples. The minimum weight loss was observed in tomatoes treated with WS5. The RS5-treated sample exhibited physiological losses of 0.43%, 1.09%, and 1.79% at 7, 15, and 30 days of storage, respectively, while the WS5-treated sample showed losses of 0.26%, 1.02%, and 1.29% over the same period. Jiang and Li (27) reported that 2% chitosan coating resulted in minimal weight loss in longan fruit. A notable difference was observed with the coating of rice and wheat straw extract. Das et al. (28) recorded a 3.53% reduction in weight loss in tomatoes coated with rice starch and coconut oil-based edible coating enriched with tea leaf extract compared to the uncoated fruits during storage at 24 °C for 20 days, demonstrating the moisture barrier properties of lipid-based edible coatings.

**Table 2:** Physiological loss of weight (%) of tomato using different concentrations of rice straw extract (RS).

**Treatments**	**Physiological loss of weight (%)**		
	**Days after treatment**		
	**7**	**15**	**30**
Control	2.31 ± 0.102^d^	3.62 ± 0.116^e^	5.42 ± 0.035^f^
RS1	2.19 ± 0.055^d^	3.23 ± 0.045^d^	5.11 ± 0.065^e^
RS2	1.86 ± 0.050^c^	2.53 ± 0.061^cd^	4.19 ± 0.047^de^
RS3	1.23 ± 0.075^b^	2.46 ± 0.051^c^	3.87 ± 0.031^c^
RS4	1.02 ± 0.032^b^	1.79 ± 0.026^b^	3.11 ± 0.025^b^
RS5	0.43 ± 0.099^a^	1.09 ± 0.041^a^	1.79 ± 0.038^a^
C.D @5%	0.24	0.20	0.15

RS, rice straw.

Values are mean ± standard deviation (n = 3) by a one-way ANOVA with Duncan's multiple range test (a, b, c, d, e, and f superscripts showed significant differences).

**Table 3:** Physiological loss of weight (%) of tomato using different concentrations of wheat straw extract (WSE).

**Treatment**	**Physiological loss of weight (%)**		
	**Days after treatment**		
	**7**	**15**	**30**
Control	2.31 ± 0.11^f^	3.62 ± 0.12^e^	5.42 ± 0.03^ef^
WS1	2.03 ± 0.04^e^	2.76 ± 0.14^d^	4.76 ± 0.08^e^
WS2	1.52 ± 0.06^de^	2.23 ± 0.04^bc^	4.03 ± 0.08^d^
WS3	1.18 ± 0.02^c^	2.19 ± 0.05^b^	3.52 ± 0.06^cd^
WS4	0.83 ± 0.09^b^	1.22 ± 0.07^ab^	2.86 ± 0.03^b^
WS5	0.26 ± 0.03^a^	1.02 ± 0.03^a^	1.29 ± 0.09^a^
C.D @5%	0.21	0.29	0.17

WS, wheat straw.

Values are mean ± standard deviation (n = 3) using a one-way ANOVA with Duncan's multiple range test (a, b, c, d, e, and f superscripts showed significant differences).

**Figure 2:**
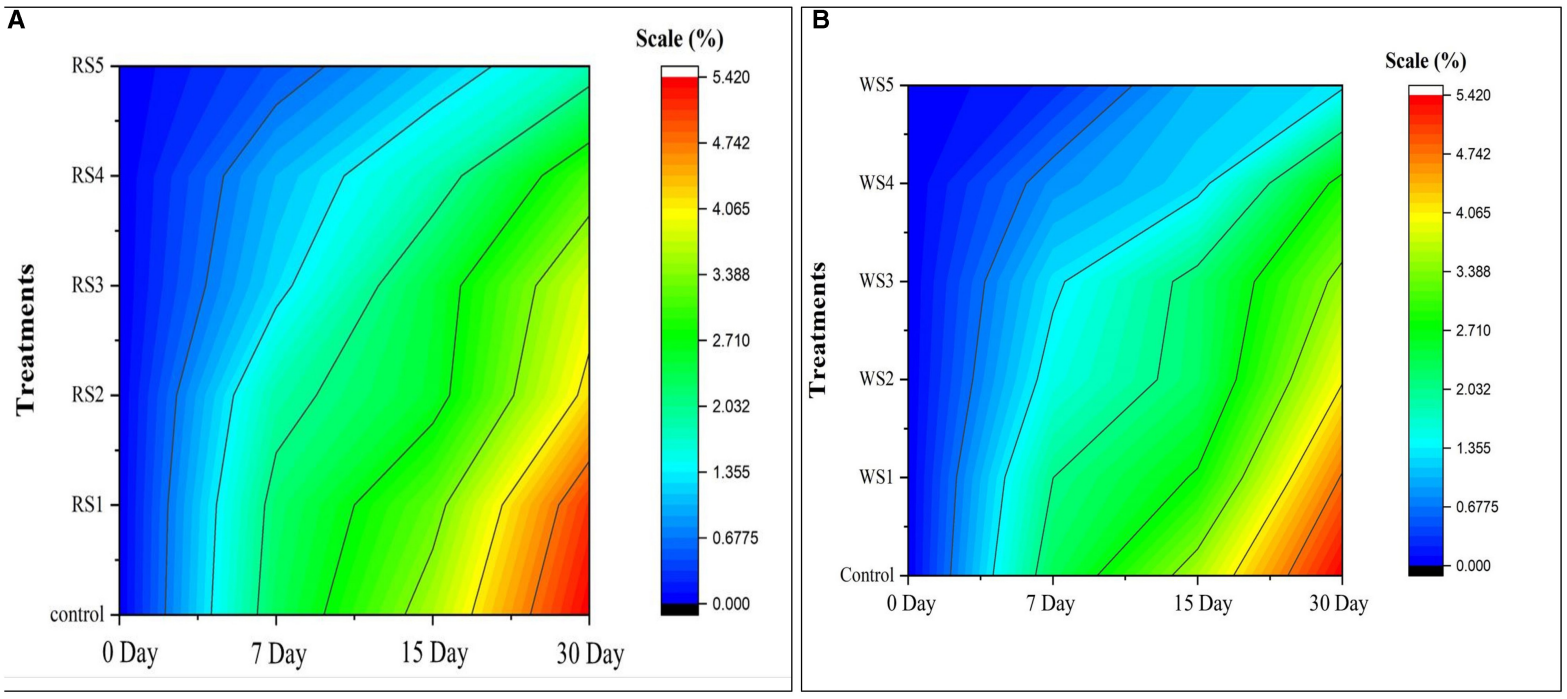
Contour map on physiological loss of weight (%) of control and treated tomatoes with different concentrations of rice straw **(A)** and wheat straw **(B)** extract.

Similarly, the color change in the contour map of tomatoes (Figure 2) depicts the change in physiological loss in weight (%) of treated and control samples of tomatoes during the storage period. Initially, on day 0, there was no change in physiological weight as indicated by the dark blue. As the storage period increased, a change in color was observed from blue to red. The red color indicates the maximum reduction in weight.

The incorporation of chitosan with different concentrations of phenolic extract affects the structural integrity of the coating, potentially increasing the number of pores through which moisture may be more readily lost (29, 30). Weight loss in fresh produce is a critical indicator of postharvest life, as it directly reflects the extent of water loss, respiration, and overall quality degradation. In this study, the storage environment and concentration of extract significantly influenced the transpiration and respiration rates of Hisar Arun tomato fruits, directly impacting their overall quality.

Similar results were observed in previous studies that indicated coatings of biopolymers on pepper (31), beeswax on carrot (80), carvacrol on tomatoes (85), cassava starch and citrus pectin on mango (75), tara gum and lipid on strawberry (83), and whey protein with oregano extract on guava (77) reduce the rate of respiration, delay senescence, and reduce the loss of texture and overall extend the shelf life of fruits and vegetables.

### 3.2 Effect of coating on firmness of tomatoes

**Figure 3:**
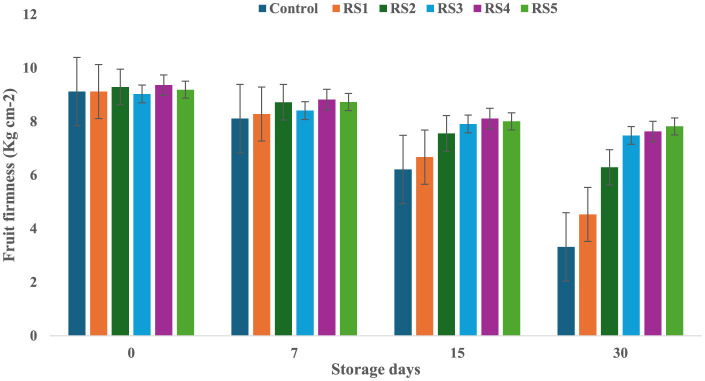
Change in fruit firmness of tomatoes during storage with coating of rice straw (RS) extract in different concentrations and uncoated tomatoes [values are mean ± standard deviation (*n* = 3)].

**Figure 4:**
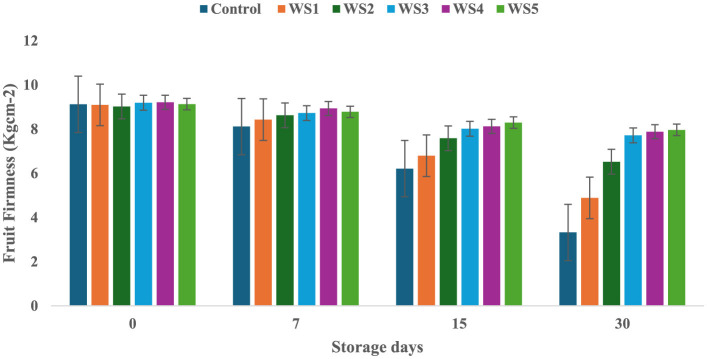
Change in fruit firmness of tomatoes during storage with coating of wheat straw (WS) extract in different concentrations and uncoated tomatoes [values are mean ± standard deviation (*n* = 3)].

On day 0 of storage, control and treated tomatoes exhibited similar firmness values, which gradually decreased over 7, 15, and 30 days, as shown in Figures 3, 4. Tomatoes treated with varying concentrations of RS and WS extract coating solution consistently showed significantly (*p* ≤ 0.05) higher firmness values as compared to the control samples. Out of all the treated tomatoes, WS5-coated tomatoes showed higher firmness values as compared to those coated with RS5. During storage, the fruit firmness gradually decreases due to metabolic and physiological processes, including the activity of the polygalacturonase enzyme, which contributes to cell wall softening (32).

A similar mechanism for firmness retention was reported by Donjio et al. (88) in tomatoes coated with pineapple peel extract and Arabic gum, which was attributed to the antioxidants present in the pineapple peel extract. In addition, coating materials act as semipermeable barriers that modify the internal atmosphere by reducing oxygen levels and increasing carbon dioxide levels, thereby slowing biochemical reactions and contributing to the preservation of fruit firmness during storage (33). The findings of the present study are consistent with those of Kumar et al. (34), who observed that tomatoes coated with a chitosan–pullulan composite edible coating enriched with pomegranate peel extract maintained higher firmness than the uncoated control during storage at 23 °C for 15 days. The coatings were effective in regulating the respiration rate of the products during postharvest storage, which, in turn, slowed down the ripening process and helped maintain fruit firmness (35). The firmness of fruits is primarily influenced by two key factors: the structural integrity of the cell wall and the turgor pressure within the fruit cells (36). Similarly, the coating of extract of aloe vera and sodium alginate increases the fruit firmness of strawberry during storage (76). A similar study by Tilahun (37) showed that mature green fruits have higher firmness values than those at the fully ripe stage. Fruit firmness typically declines when cell wall integrity is compromised or turgor pressure decreases. In contrast, the minimum water loss was observed in tomatoes treated with RS and WS extracts, i.e., this helped to preserve the cell wall integrity and maintain higher turgor pressure, ultimately leading to improved firmness of fruits (33). By minimizing water loss, the bioactive edible coating played a crucial role in maintaining the structural integrity of the fruit, resulting in a more stable and firmer texture.

### 3.3 Effect of coating on pH of tomatoes

**Figure 5:**
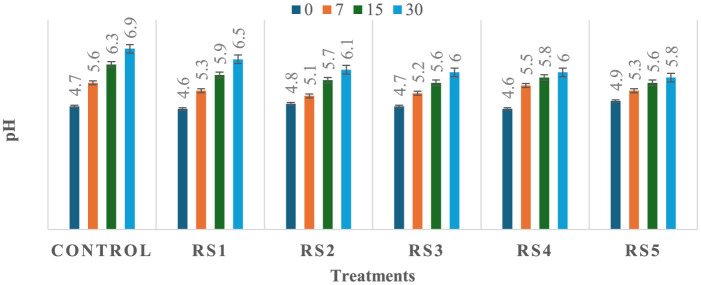
Change in pH of tomatoes during storage with coating of rice straw (RS) extract in different concentrations and uncoated tomatoes [values are mean ± standard deviation (*n* = 3)].

**Figure 6:**
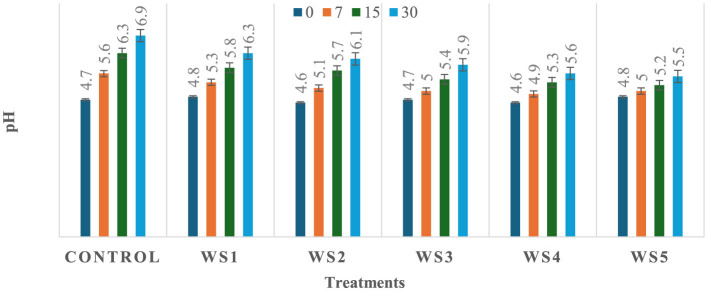
Change in pH of tomatoes during storage with coating of wheat straw (WS) extract in different concentrations and uncoated tomatoes [values are mean ± standard deviation (*n* = 3)].

The observed variation in pH among the treated and untreated samples of tomatoes could be attributed to differences in metabolic processes and their interactions with the applied coating materials, which may influence the rate of respiration and biochemical activities in the fruit, leading to a change in pH. As shown in Figures 5, 6, a significant (*p* ≤ 0.05) difference in pH values was observed among the stored tomato samples. The lowest pH values, 5.5 and 5.8, were recorded in WS5- and RS5-treated samples after 30 days of storage, whereas the highest pH value, 6.9, was obtained in the control sample of tomatoes. This suggests that the coating materials had a significant impact on the pH levels of the tomatoes, with the WS5 and RS5 treatments resulting in a more acidic environment.

A decrease in pH values may be associated with an increase in the titrable acidity of the tomato fruits, potentially resulting from a lowered respiration rate that slows down the breakdown and consumption of organic acids during storage (27). As a result, the organic acids accumulate, leading to a decrease in pH values and an increase in titrable acidity. Several studies have reported that as tomato fruits transition from the mature-green stage to full ripeness, their pH tends to increase due to the degradation of organic acids during the ripening process (38, 39, 79). Ribeiro-Santos et al. (40) reported a slight increase in pH (from 4.62 to 5.77) in tomatoes coated with cassava starch–chitosan coatings enriched with *Lippia sidoides* essential oils and pomegranate peel extract during storage at 25 °C for 12 days, compared to the uncoated control. The pH results of the present study are also consistent with those of Firdous et al. (41), who observed a slight increase from 4.98 to 5.00 in tomatoes coated with 80% aloe vera gel and 2% calcium chloride after 30 days of storage. Fruits coated with chitosan have exhibited lower acidity loss, and similar observations were also found in previous studies conducted on strawberry (42, 84), guava and litchi (43), and capsicum (73).

### 3.4 Change in titrable acidity (TA) of tomatoes during storage

**Figure 7:**
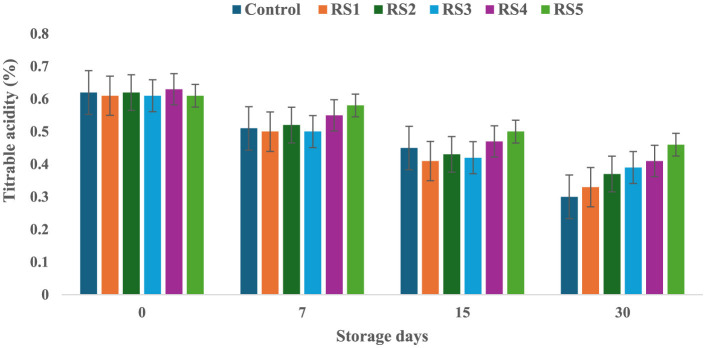
Change in titrable acidity of tomatoes as a function of storage time for coated tomatoes with rice straw (RS) extract in different concentrations (RS1: 0.2, RS2: 0.4, RS3: 0.6, RS4: 0.8, and RS5-1%) and uncoated tomatoes (control) [values are mean ± standard deviation (*n* = 3)].

**Figure 8:**
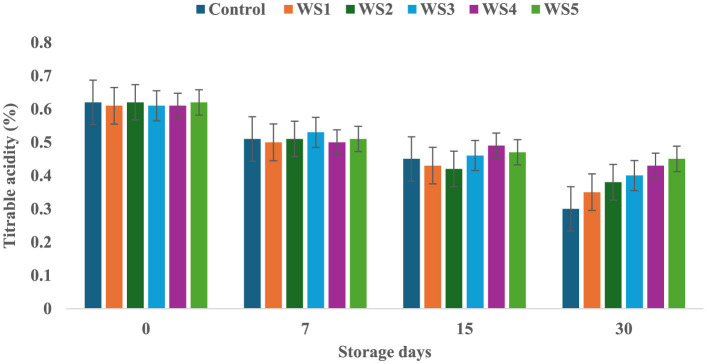
Change in titrable acidity of tomatoes as a function of storage time for coated tomatoes with wheat straw (WS) extract in different concentrations (WS1: 0.2, WS2: 0.4, WS3: 0.6, WS4: 0.8, and WS5: 1%) and uncoated tomatoes (control) [values are mean ± standard deviation (*n* = 3)].

The titrable acidity of tomatoes was significantly affected by the different concentrations of extract during the storage for 30 days. The titrable acidity of the untreated or control sample of tomatoes was significantly (*p* < 0.05) decreased from 0.62 to 0.30 during the 30 days of storage, but in the treated samples, the titrable acidity was consistently decreased during the storage days. The RS5-treated samples showed a minimum (0.46) decrease in titrable acidity, followed by RS4 (0.41) and RS3 (0.39) (Figure 7). Similarly, the WS5-treated sample showed a minimum (0.45) decrease in titrable acidity, followed by WS4 (0.43) and WS3 (0.40) (Figure 8).

A higher loss of titrable acidity in tomatoes is often associated with increased respiration and ripening rates, as organic acids are utilized as substrates in the respiration process (44). Adjouman et al. (45) reported a significant delay in titrable acidity (TA) changes in tomatoes coated with cassava starch-based composite edible coatings compared to both uncoated fruits and those coated with commercial Semperfresh™. Moreover, another study by Saliba-Colombani et al. (46) had shown that total sugars were positively correlated to pH and titrable acidity. Additionally, studies have found a positive correlation between total sugars and pH/titrable acidity, indicating that fruits with higher sugar content tend to have more free organic acids and lower hydrogen ion concentrations. This suggests that fruits with higher sugar content may have a more acidic pH. Furthermore, it has been observed that titrable acidity content generally decreases during ripening and storage (47). However, maintaining higher fruit acidity is beneficial, as it can reduce the incidence of fungal infection (48). Similar results were also reported by Tigist et al. (87), who observed a general trend of increasing ascorbic acid content during the early ripening stages, followed by a decline at full ripeness of tomatoes. Likewise, Ali et al. (90) found that papaya fruits coated with chitosan exhibited a slower initial increase in ascorbic acid compared to uncoated fruits.

### 3.5 Effect of coating on color of tomatoes

**Figure 9:**
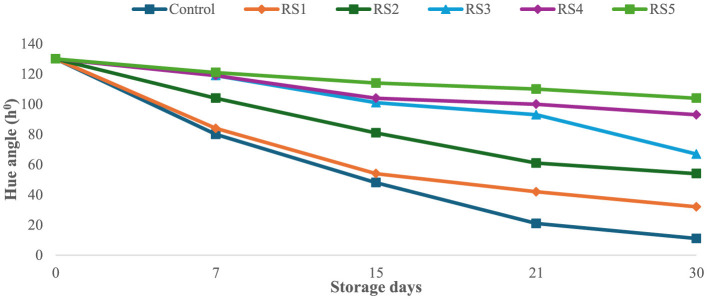
Effect of coating of rice straw (RS) extract on the hue angle of tomatoes during storage.

**Figure 10:**
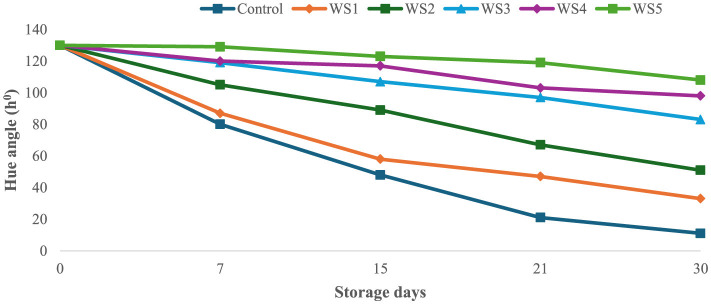
Effect of coating of wheat straw (WS) extract on the hue angle of tomato fruit during storage.

The bioactive coating formed a thin, homogeneous, semipermeable membrane on the surface of the tomato skin, facilitating controlled gas exchange. Throughout the experimental storage period, noticeable changes were observed in the L^*^, a^*^, and b^*^ values of all the tomato samples. The L^*^ values remained stable until the ripening stage, indicating that brightness remained constant, while the tomatoes were green. The L^*^ gradually decreased progressively with increasing storage time of untreated and treated tomato samples. However, this decrease was slower for tomatoes treated with 1.0 g/mL phenolic extract of rice straw and wheat straw as compared to untreated samples. After 15 days of storage, tomatoes treated with RS5 and WS5 maintained significantly higher chroma values compared to other treated and control tomatoes, indicating better color retention. However, the color was significantly (*p* < 0.05) changed in uncoated samples as compared to the treated samples of tomatoes. The values were sharply decreased in the control after the 7th day of storage, as compared to the treated samples of tomatoes. The hue angle remained stable in RS2-, RS3-, RS4-, and RS5-treated samples up to 15 days (Figure 9), followed by a slight decrease thereafter. A similar pattern was observed in tomatoes treated with wheat straw extract (Figure 10). This reduction was attributed to the formation of a thick, continuous coating that covered the epidermal openings and modified the internal atmosphere, leading to elevated carbon dioxide and reduced oxygen levels (49).

According to Paul et al. (49), tomatoes coated with 2.15% chitosan and 0.05% glycerol exhibited a reduced respiration rate of 21.21 ± 0.06 mg CO_2_ kg^−1^ h^−1^ and a DE of 2.31 ± 0.01 during storage. This reduction was attributed to the formation of a thick and continuous coating that covered the epidermal openings, thereby modifying the internal atmosphere by increasing carbon dioxide levels and reducing oxygen availability.

### 3.6 Effect of coating on lycopene content of tomatoes during storage

**Figure 11:**
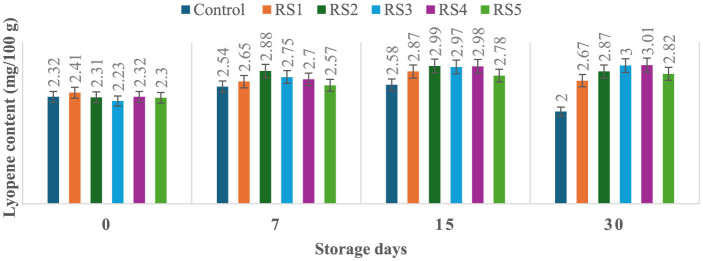
Effect of coating of rice straw extract on lycopene content of tomatoes during storage (RS1: 0.2, RS2: 0.4, RS3: 0.6, RS4: 0.8, and RS5: 1%) [values are mean ± standard deviation (*n* = 3)].

**Figure 12:**
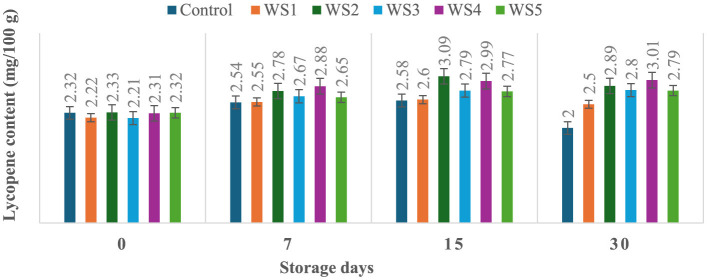
Effect of coating of wheat straw extract on lycopene content of tomatoes during storage (WS1: 0.2, WS2: 0.4, WS3: 0.6, WS4: 0.8, and WS5-1%) [values are mean ± standard deviation (*n* = 3)].

The lycopene content of tomatoes varied with the ripening stages, and similar variations were observed in the experimental setup. Ripeness is characterized by a decline in chlorophyll concentration and a rapid production of red pigment lycopene. On day 30 of storage, the lycopene content of control tomato samples during their ripening stage was 2 mg/100 g. In contrast, the lycopene content of rice straw-coated samples (RS1, RS2, RS3, RS4, and RS5) was 2.67, 2.87, 3.00, 3.01, and 2.81 mg/100 g, respectively. For wheat straw extract-coated samples (WS1, WS2, WS3, WS4, and WS5, the lycopene content was 2.50, 2.89, 2.80, 3.01, and 2.79 mg/100 g, respectively. Both treated and control tomatoes showed a consistent rise in lycopene content until the 15 days of storage, followed by a gradual decline. However, tomatoes treated with wheat straw extract exhibited a steady increase in lycopene content throughout the entire 30-day storage period. The earlier increase in lycopene content in tomato fruits may be due to the faster ripening, which facilitates the accumulation of lycopene. During this process, lycopene integrates into the internal cell membranes, and the conversion of chloroplasts into chromoplasts occurs (81). Figures 11 and 12 illustrate the changes in lycopene content for tomatoes treated with varying concentrations of rice straw and wheat straw extracts over the 30-day storage period.

Javanmardi and Kubota (50) reported that temperature range and respiration rate are the major factors influencing lycopene synthesis in tomatoes during storage. Ali et al. (51) reported that lycopene content generally increased with the storage time in both treated and control fruits. However, tomatoes treated with high concentrations of gum arabic (15% and 20 %) exhibited the lowest lycopene levels, even after 20 days of storage. Furthermore, it has been noted that the rate of transpiration during storage significantly influences lycopene synthesis (50). Coatings reduce respiration by limiting oxygen exposure. After 15 days, lycopene content in red-ripe fruits was 0.95, 0.59, and 0.62 mg/100 g, for control, chitosan, and pectin-coated samples, respectively (52). Ripening-related pigment changes are characterized by the conversion of chloroplasts into chromoplasts, leading to a rapid accumulation of carotenoids, particularly lycopene, and a concurrent decline in chlorophyll content (53). These transformations underline the dynamic changes in tomato pigmentation during ripening and storage.

### 3.7 Effect of coating on β-carotene content of tomatoes during storage

**Figure 13:**
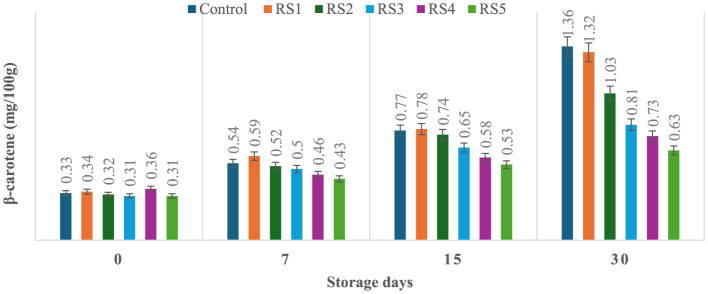
Effect of coating of rice straw extract on β-carotene content of tomatoes during storage (RS1: 0.2, RS2: 0.4, RS3: 0.6, RS4: 0.8, and RS5: 1%) [values are mean ± standard deviation (*n* = 3)].

**Figure 14:**
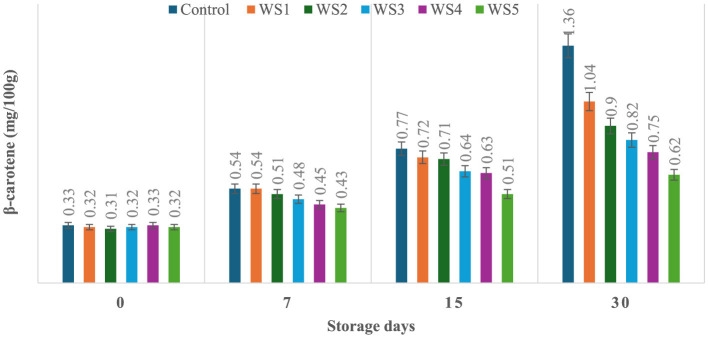
Effect of coating of wheat straw extract on β-carotene content of tomatoes during storage (WS1: 0.2, WS2: 0.4, WS3: 0.6, WS4: 0.8, and WS5: 1%) [values are mean ± standard deviation (*n* = 3)].

The production of β-carotene was significantly (*p* ≤ 0.05) higher in the untreated or control sample of tomatoes as compared to the treated tomato samples stored for 30 days. Control, RS1-, RS2-, RS3-, RS4-, and RS5-treated samples showed 0.33, 0.34, 0.32, 0.31, 0.36, and 0.31 mg/100 g beta-carotene content on day 0 after coating. For untreated samples of tomatoes, the content was increased to 1.36 mg/100g after 30 days of storage, whereas in treated samples, it consistently was increased to 1.32, 1.03, 0.81, 0.73, and 0.63 for RS1, RS2, RS3, RS4, and RS5, respectively (Figure 13). Similarly, in the WS5-treated tomatoes, minimum beta-carotene content (0.62 mg/100 g) was observed at 30 days of storage, followed by WS4- and WS3-treated tomatoes (Figure 14).

The ripening process in tomatoes is primarily characterized by the production and accumulation of pigments, particularly carotenoids such as lycopene (54, 55). This study proposed that a higher concentration of bioactive compounds in the coating can act as a barrier to ethylene gas and slow down the ripening process. As a result, the control samples might exhibit higher beta-carotene levels than the treated tomato samples.

### 3.8 Effect of coating on ascorbic acid content of tomatoes during storage

**Figure 15:**
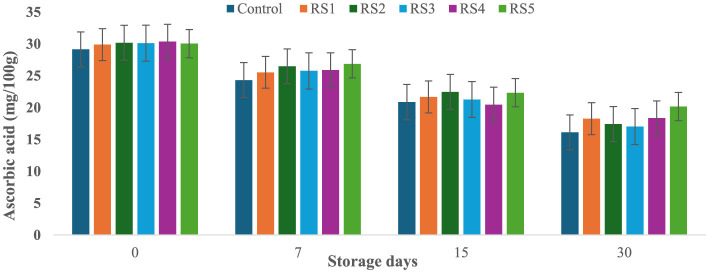
Effect of coating of rice straw extract on ascorbic acid content of tomatoes during storage (RS1: 0.2, RS2: 0.4, RS3: 0.6, RS4: 0.8, and RS5-1%) [values are mean ± standard deviation (*n* = 3)].

**Figure 16:**
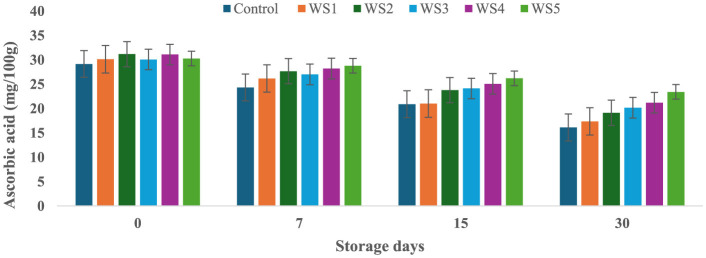
Effect of coating of wheat straw extract on ascorbic acid content of tomatoes during storage (WS1: 0.2, WS2: 0.4, WS3: 0.6, WS4: 0.8, and WS5-1%) [values are mean ± standard deviation (*n* = 3)].

At the start of the experiment, the ascorbic acid content in the control tomato samples was 29.14 mg/100 g, which rapidly decreased to 16.11 mg/100 g after 30 days of storage. In contrast, tomatoes treated with varying concentrations of rice straw and wheat straw extracts exhibited a more gradual reduction in ascorbic acid content over the same period. Figures 15 and 16 illustrate the effect of these bioactive coatings on the ascorbic acid levels of tomatoes during storage. Among the treated samples, the minimum reduction in ascorbic acid content was observed in RS4- and RS5-treated tomatoes, followed by RS3 and RS2. A similar trend was noted in tomatoes treated with wheat straw extract, with higher concentration treatments (WS4 and WS5) showing the most significant retention of ascorbic acid content.

This demonstrates that the bioactive coatings from rice and wheat straw extracts effectively slow the degradation of ascorbic acid, preserving the nutritional quality of the tomatoes during storage. Bioactive coatings from rice and wheat straw extracts help preserve ascorbic acid in tomatoes by forming semipermeable barriers that reduce oxygen diffusion and respiration, thereby slowing oxidative degradation (52). The phenolic compounds present in these extracts further scavenge reactive oxygen species and inhibit oxidative enzymes, stabilizing the ascorbate pool (33, 71). In addition, reduced transpiration maintains tissue integrity, which contributes to slower vitamin C loss during storage (56, 57). The observed pattern aligns with general trends, where fruits and vegetables experience ascorbic acid depletion with prolonged storage and higher temperatures (58, 59). Similar reductions in ascorbic acid content during storage have been reported in bananas (60), tomatoes (61), and kiwifruit (62).

### 3.9 Diseases incidence in tomatoes during the storage period

**Figure 17:**
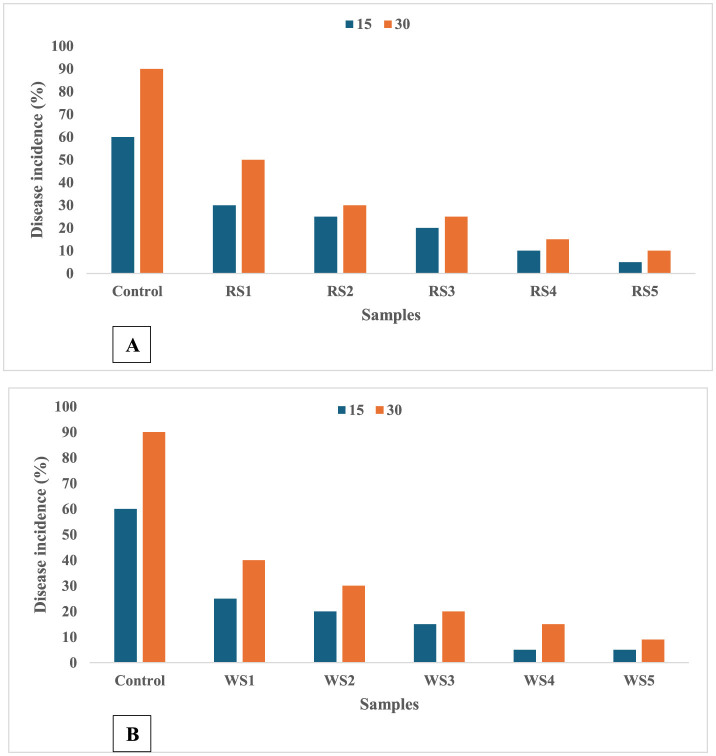
Effect of coating of rice straw **(A)** and wheat straw **(B)** extract on disease incidence of tomatoes during storage of 15 and 30 days (RS, rice straw; WS, wheat straw).

A significant difference was observed in the frequency of disease occurrence in tomato fruits throughout the storage period. The coating application postponed the rate of firmness loss by preserving the integrity of the cell wall. Moreover, the coating could lower the rate of ethylene synthesis and the metabolic pathway of respiration (63). When these circumstances come together, cell walls may be better able to withstand fungal invasion (64). Figures 17A, B illustrate that disease incidence in tomato fruits was significantly influenced by the different coating treatments of rice and wheat straw extract. Coated tomatoes exhibited a marked reduction in disease occurrence compared to untreated control fruits. The control sample of tomatoes showed a 90% incidence of disease after 30 days of storage, while the RS5- and WS5-treated samples showed lowest disease incidence. This demonstrates the effectiveness of the coatings in mitigating disease progression during storage.

### 3.10 Disease severity in tomatoes during storage

**Figure 18:**
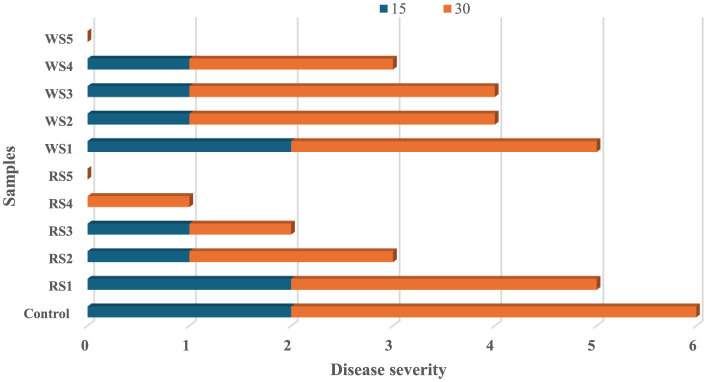
Effect of coating on disease severity of tomatoes during storage of 15 and 30 days (RS, rice straw; WS, wheat straw).

The impact of different coatings on the disease severity of tomatoes during storage of 15 and 30 days is shown in Figure 18. No diseases were observed in the treated tomato sample on the 7th day of storage, whereas the untreated or control samples exhibited rot on the 7th day. On 15 days of storage, control samples showed a disease severity of 2, while tomatoes treated with rice straw extract coatings—RS1, RS2, RS3, RS4, and RS5—exhibited disease severity levels of 2, 1, 1, 1, and 0, respectively. On day 30, tomatoes treated with RS4 and RS3 had disease severity levels of 1 and 0, respectively. Similarly, tomatoes treated with wheat straw extract (WS)—WS1, WS2, WS3, WS4, and WS5—displayed disease severity levels of 3, 3, 3, 2, and 0, respectively.

The bioactive coatings significantly enhanced the cellular and tissue integrity of fresh tomatoes, reducing their susceptibility to pathogenic infections (64). This protective effect was particularly evident as the tomatoes approached senescence, a process that the coatings helped to delay.

### 3.11 Shelf life of tomatoes

After harvesting, the time taken by fruits to begin deteriorating is considered their shelf life (65). The lowest shelf life of 10 days recorded for uncoated fruits was likely due to accelerated physiological changes and metabolic activities, including increased respiration and ethylene biosynthesis during storage, which led to fruit senescence (66, 67). At the senescence stage, fruits become more susceptible to microbial infections because of the loss of cellular and tissue integrity, resulting in rapid deterioration (68). In contrast, fruits coated with formulations of 1.0% extracts of rice and wheat straw exhibited a marked extension of shelf life by 17 and 15 days, respectively, at 25 ± 2 °C and 75 ± 5% RH. This extension can be attributed to the coatings' ability to reduce respiration rate, ethylene production, physiological changes, microbial decay, and senescence. Extending the shelf life of tomatoes offers significant economic benefits by reducing postharvest losses, enabling wider market access, improving retail efficiency, increasing revenue, enhancing consumer satisfaction, and promoting sustainability. Collectively, these advantages contribute to a stronger and more efficient supply chain, benefiting all stakeholders involved (52, 69, 70).

## 4 Conclusion

This study demonstrated that bioactive coatings enriched with rice and wheat straw extracts significantly influenced the postharvest quality and shelf life of tomatoes. The coatings formed a thin, semipermeable membrane on the tomato surface, effectively reducing weight loss, preserving firmness, and delaying color changes during storage. Tomatoes treated with higher concentrations of RS and WS extracts, particularly RS4, RS5, WS4, and WS5, exhibited improved preservation of physicochemical properties, including ascorbic acid and lycopene content. These bioactive compounds contributed to the antioxidant and antimicrobial properties of the coatings, which enhanced the cellular and tissue integrity of the fruits and reduced disease incidence and severity during storage. The results also indicated that while the coatings slowed down ripening processes, overconcentration of bioactive compounds could decrease film flexibility, potentially reducing coating efficacy. Despite this, RS5- and WS5-treated tomatoes maintained superior quality compared to control samples, exhibiting reduced disease severity and better retention of chroma and firmness over 30 days of storage. These findings suggest that the application of bioactive coatings derived from agricultural waste extracts (rice straw and wheat straw) offers a sustainable and effective solution for extending the shelf life and maintaining the postharvest quality of fresh tomatoes. This approach not only adds value to agricultural byproducts but also provides an eco-friendly alternative for reducing postharvest losses in fruits. Transforming waste from straw into food packaging can offer two significant advantages: (i) decreased environmental impact and lower disposal costs associated with both plastic packaging and agricultural waste and (ii) extended shelf life for food and reduced food waste. Additional research is needed to evaluate the effectiveness of bioactive packaging in preserving the quality of various food product categories under different storage conditions.

## Data Availability

The datasets presented in this article are available on request. Requests to access the datasets should be directed to Kamla Malik via kamlamalik06@gmail.com.
